# The Emerging World of Microbiome in Autoimmune Disorders: Opportunities and Challenges

**DOI:** 10.4103/injr.injr_210_20

**Published:** 2021-03-23

**Authors:** Ashutosh K. Mangalam, Meeta Yadav, Rajwardhan Yadav

**Affiliations:** 1 Department of Pathology, University of Iowa, Iowa, IA, USA; 2 Department of Rheumatology, St Francis Hospital, Hartford, CT, USA

**Keywords:** Autoimmunity, gut, inflammation, microbiome, T cells, T-helper 17, regulatory T

## Abstract

Trillions of commensal bacteria colonizing humans (microbiome) have emerged as essential player(s) in human health. The alteration of the same has been linked with diseases including autoimmune disorders such as multiple sclerosis, rheumatoid arthritis, systemic lupus erythematosus, and ankylosing spondylitis. Gut bacteria are separated from the host through a physical barrier such as skin or gut epithelial lining. However, the perturbation in the healthy bacterial community (gut dysbiosis) can compromise gut barrier integrity, resulting in translocation of bacterial contents across the epithelial barrier (leaky gut). Bacterial contents such as lipopolysaccharide and bacterial antigens can induce a systemic inflammatory environment through activation and induction of immune cells. The biggest question in the field is whether inflammation causes gut dysbiosis or dysbiosis leads to disease induction or propagation, i.e., it is inside out or outside in or both. In this review, we first discuss the microbiome profiling studies in various autoimmune disorders, followed by a discussion of potential mechanisms through which microbiome is involved in the pathobiology of diseases. A better understanding of the role of the microbiome in health and disease will help us harness the power of commensal bacteria for the development of novel therapeutic agents to treat autoimmune disorders.

## Introduction

Autoimmune disorders are characterized by immune-mediated inflammatory processes which lead to target organ damage, resulting in pathological states that can culminate in significant morbidity and mortality. Inflammation is a highly evolved process in vertebrates and higher animals that enable the host to tackle microbial and foreign insults. Through the process of inflammation, immune cells attempt to eliminate infectious organisms from the host. However, the same inflammatory process can be associated with the development of autoimmune or inflammatory disorders. The precise mechanism(s) leading to the initiation of inflammatory processes and further development of autoimmune disorder is not well understood, but it is widely accepted that genetic and environmental factors predispose to the development of these disorders. Majority of genes linked with autoimmune disorders are immune-related genes, with *HLA* genes displaying the strongest linkage with disease predisposition/protection. However, limited concordance in the development of autoimmune disorders in monozygotic twins suggests the requirement of additional risk variables. Besides the host genes, environmental factors have been linked with the pathogenesis of autoimmune diseases. Despite numerous environmental factors being proposed over the years, no single factor has garnered significant strength in influencing outcomes in autoimmune disorders. As host immune responses play a key role in autoimmune disease pathogenesis, environmental factors with their ability to influence host immune responses appear to be intriguing candidate(s). In recent years, microbiome has gained a pivotal role as potential environmental factor influencing immune responses.^[1]^ Microbiome consists of trillions of microbes, especially bacteria, viruses, and fungi, residing on and inside human body. Ability of host microbiome predominantly at the mucosal surfaces to regulate local as well as systemic host immune responses makes microbiome a critical environmental factor contributing to predisposition, progression, and also protection of autoimmune disorders.^[2]^ Recent landmark studies have highlighted that alterations in the gut microbial community influences disease outcomes in a variety of autoimmune disorders, thus further endorsing the vital role of host microbiome in influencing autoimmunity.^[2–11]^ Human gut is colonized by a large number of microorganisms (bacteria, viruses, and fungi) that support various physiologic functions.^[12,13]^ Healthy individuals have a diverse repertoire of beneficial commensals (symbionts), which assist in maintaining homeostasis within the gut. These symbionts process undigested food, extract nutrients, and generate beneficial metabolites, which are involved in maintaining the healthy state in the gut. In addition, these microbes through multiple mechanisms maintain integrity of the gut mucosal barrier, resist growth of pathogens, and regulate host immune responses. It is hypothesized that perturbation in the healthy gut microbiome (gut dysbiosis) leads to the development and exacerbations of various autoimmune disorders, such as rheumatoid arthritis (RA), ankylosing spondylitis (AS), systemic lupus erythematous (SLE) disorders, multiple sclerosis (MS), juvenile idiopathic arthritis, reactive arthritis, systemic sclerosis, and type 1 diabetes (T1D). A number of factors, including genetics, diet, modern lifestyles, environmental toxins, and stringent hygienic measures, can induce gut dysbiosis by tilting the balance between symbionts and bacteria that can disrupt normal homeostasis (pathobionts) toward an inflammatory phenotype.^[14,15]^ The mechanisms through which gut dysbiosis can predispose individuals to autoimmune disorders are being actively pursued by researchers across the globe.

In this review, we first discuss microbiome association studies with various autoimmune disorders in humans and in animal models. In the later part of this article, we discuss potential mechanisms through which gut microbiome can influence disease outcomes in inflammatory disorders and potential utilization of bacteria as drugs for various autoimmune diseases.

## Microbiome Associations with Autoimmune Disorders

### Multiple sclerosis

Microbiome studies across varying geographic regions (USA, Japan, UK, and Italy) have highlighted that MS patients have a distinct microbiome signature compared to healthy controls (HCs).^[6,8,14,16–18]^ These studies have shown enrichment or depletion of specific bacterial genera when compared to HCs. We have summarized MS microbiome studies in a recent review from our group [Table 1]. Although there is some variability in microbiome signatures, among different MS studies, certain bacterial abundances were similar across multiple studies. We reported loss of certain genera such as *Prevotella*, *Parabacteroides*, *Adlercreutzia*, and *Lactobacillus* in relapsing and remitting multiple sclerosis (RRMS) patients [Table 1].^[6]^ Other groups have also shown loss of one or more of these bacteria in their RRMS cohorts.^[6,8,14,16–18,20]^ Multiple groups have reinforced that *Prevotella* is in lower abundance in RRMS patients.^[6,8,18,20]^ Besides our group, Miyake *et al*., Jangi *et al*., and Cosorich *et al*. showed lower levels of *Prevotella* in RRMS patients.^[6,8,18,20]^ Among these, the study by Cosorich *et al*.^[20]^ is significant as it has shown an inverse correlation between *Prevotella* abundance and MS disease severity. In this study, duodenal biopsies of MS patients with severe disease had relatively lower levels of *Prevotella*. In addition, the same study also showed that RRMS patients with higher levels of interleukin (IL)-17 had lower levels of *Prevotella.* Thus, collectively, these studies imply that an increased abundance of *Prevotella* is associated with healthier or milder disease phenotype, plausibly due to dampening of proinflammatory processes.

Besides *Prevotella*, other bacteria such as *Parabacteroides* and *Adlercreutzia* have also been shown to have lower abundance when compared to HCs. Cekanaviciute *et al*.^[17]^ reported lower abundance of *Parabacteroides distasonis* in the treatment-naive RRMS patients compared to HCs, suggesting a potentially protective effect of *P. distasonis* in RRMS.^[17]^ Berer *et al*.^[16]^ showed that germ-free (GF) mice transplanted with fecal matters from HC showed higher abundance of *Adlercreutzia* as compared to mice receiving fecal transplant from RRMS patients.^[16]^ Interestingly, all these bacteria (*Prevotella*, *Parabacteroides*, and *Adlercreutzia*) can metabolize phytoestrogen into beneficial metabolites. The importance of phytoestrogen metabolites and gut bacteria in human health is an area of active research which is discussed in a recent review from our group.^[21]^ *Firmicutes* such as *Akkermansia*, *Dorea*, and *Archaea-Methanobrevibacter* have been shown to be enriched in stool from RRMS patients,^[6,8,17]^ suggesting that these gut microbes might have proinflammatory effects [Table 1]. We have recently analyzed fecal microbiome from MS patients in Iowa, and besides *Akkermansia* and *Dorea*, we have also observed an increase in *Eggerthella* spp. (unpublished observation). Collectively, MS microbiome studies suggest that loss of *Prevotella*, *Parabacteroides*, *Adlercreutzia*, and *Lactobacillus* and/or enrichment of *Akkermansia*, *Dorea*, *Eggerthella*, and *Archaea-Methanobrevibacter* might play a role in the predisposition and/or exacerbation in RRMS. We believe that this increased abundance could reasonably contribute to the induction and/or maintenance of proinflammatory cells in the gut, thus leading to a systemic inflammatory state consistently observed in RRMS patients.

### Rheumatoid arthritis

Oral and gut microbiome studies have shown association of microbiome with RA development and progression^[5,7,22–24]^ [Table 2]. Majority of the studies have highlighted the role of oral microbiome and generation of anti-cyclic citrullinated peptide (ACPA) antibodies. However, there are a few studies that have demonstrated unique gut microbiome signature patterns in RA patients. Individuals with new-onset rheumatoid arthritis have a higher relative abundance of *Prevotella copri* at the expense of *Bacteroides* species in their gut.^[25]^ A study from Mayo Clinic, USA, highlighted that RA patients have a significant abundance of *Actinobacteria*, *Collinsella*, *Eggerthella*, and *Actinomyces* and lower levels of *Faecalibacterium* compared to HCs.^[7]^ However, this study failed to find an expansion of *P. copri* as reported in previous study by Scher and Abramson.^[25]^ An Italian study showed that treatment-naïve RA patients from Italy displayed an increased abundance of *Lactobacillales* and lower levels of *Faecalibacterium*, *Flavobacterium*, and *Blautia*.^[23]^ A comprehensive study of RA patients from China also showed expansion of *Lactobacillus* and *Eggerthella* in treatment-naïve RA patients as compared to HCs. Besides these, RA patients also showed expansion of *Clostridium asparagiforme*, *Gordonibacter pamelaeae*, and *Lachnospiraceae bacterium*.^[24]^ These authors noted that *Haemophilus* spp., *Veillonella, Klebsiella pneumoniae*, *Bifidobacterium bifidum*, *Sutterella wadsworthensis*, and *Megamonas hypermegale* had a lower abundance in RA patients compared to HCs.^[24]^ This study also failed to draw any correlation between *P. copri* and treatment-naïve RA patients. Thus, the role of *P. copri* as a potential pathogen in RA is debatable.

Besides the gut, oral and lung microbiome signatures have also been profiled in RA patients. Exacerbation of disease activity has been very well documented in patients who have poor oral hygiene and thus unique oral microbial signatures. A study from China reported that treatment-naïve patients have a unique microbiota signature during diagnosis.^[24]^ However, treatment with a disease-modifying antirheumatic drug methotrexate led to restoration of the oral microbiome signatures which were similar to oral microbiome observed in HCs.^[24]^ Another study from China showed that oral microbiome of RA patients had enrichment of *Neisseria subflava*, *Haemophilus parainfluenzae*, *Veillonella dispar, Prevotella tannerae, Actinobacillus parahaemolyticus, Neisseria, Haemophilus, Prevotella, Veillonella, Fusobacterium, Aggregatibacter,* and *Actinobacillus* species.^[5]^ A Canadian study showed enrichment of *Actinomyces, Eggerthella, Clostridium III, Faecalicoccus, Roseburia,* and *Streptococcus* in RA patients compared to HCs.^[22]^ Thus, studies across various continents (North America, Europe, and Asia) suggest that RA patients have a distinct gut and oral microbiome compared to HCs. We discuss the potential mechanisms through which microbial dysbiosis might predispose and/or propagate disease.

### Systemic lupus erythematosus

Like other autoimmune diseases, lupus patients also have gut and oral microbial dysbiosis [Table 3]. First report on gut microbiota in SLE was a study of 20 SLE patients in remission and who had not received any antibiotics or immunomodulating medications in the last 6 months.^[26]^ The study observed that *Bacteroidetes* phyla were significantly higher in the SLE patients when compared to HCs. *Firmicutes* to *Bacteroidetes* (F/B) ratio was significantly lower in SLE patients even in remission when compared to HC (*P* < 0.002). The total fecal bacteria levels were similar among the two groups. Gut dysbiosis was also reported in another study of SLE patients (*n* = 45) from China.^[27]^ In this study, authors reported that SLE patients had a higher abundance of *Rhodococcus*, *Eubacterium*, *Flavonifractor*, *Klebsiella*, and *Prevotella* and lower abundance/depletion of *Pseudobutyrivibrio* and *Dialister.*^[27]^ However, a study by Luo *et al*. failed to find lower F/B ratio in human SLE patients as reported by other studies.^[28]^ They reported that SLE patients have higher levels of phylum *Proteobacteria* and genus *Blautia* and lower levels of bacteria belonging to genus *Odoribacter* and family *Rikenellaceae.* In the largest studies of 61 female patients with lupus nephritis, Silverman *et al*. reported an enrichment of *Ruminococcus gnavus.*^[29]^ In addition, patients with active nephritis showed higher levels of *R. gnavus* which has recently been reassigned to the genus *Blautia*.^[29]^ Interestingly, levels of *Blautia* have been shown to be higher in other inflammatory autoimmune disorders such as MS, Crohn’s disease, and AS patients with a history of Inflammatory Bowel Disease (IBD).^[4,6,32]^ Enrichment of *R.* gnavus/*Blautia* in multiple autoimmune disorders points toward an inherent ability of these microbes to promote a proinflammatory environment.

Finally, oral microbiome studies from SLE patients have also confirmed microbial dysbiosis as SLE patients had distinct microbiome signature compared to HCs. In a study of 20 SLE patients and 19 healthy controls, Li *et al*.^[31]^ showed an enrichment of bacteria belonging to families *Lactobacillaceae*, *Veillonellaceae*, and *Moraxellaceae* and depletion of *Sphingomonadaceae*, *Halomonadaceae*, and *Xanthomonadaceae* families. In addition, Corrêa *et al*. reported that SLE patients had higher prevalence of periodontitis compared to HCs with higher bacterial load and reduced bacterial diversity.^[30]^ *Fretibacterium, Prevotella nigrescens,* and *Selenomonas* were present at higher abundance in SLE patients compared to HCs. The presence of certain oral bacteria such as *Veillonella* species in the gut suggests that oral dysbiosis can lead to the translocation of bacteria from oral cavity to the intestine.

### Ankylosing spondylitis

The unique microbial signature has been also reported in patients with AS from the terminal ilea by Breban *et al*. when compared to HCs.^[4]^ A significant abundance of *Lachnospiraceae*, *Veillonellaceae*, *Prevotellaceae*, *Porphyromonadaceae*, and *Bacteroidaceae* was observed in patients with AS.^[33]^ In a recent study, Breban *et al*. [4] demonstrated that microbial dysbiosis was present in patients with spondyloarthropathy (SpA) as well as RA and the microbiome signatures were disease specific. In particular, a two-fold to three-fold increase in the abundance of *R. gnavus* was observed in patients with SpA, as compared with patients with RA and HCs.^[4]^ Recent study noted that patients with AS demonstrated an increase in the abundance of *Prevotella melaninogenica*, *P. copri*, and *Prevotella* sp. and a decrease in the abundance of *Bacteroides* spp.^[34]^ Altered microbiota, characterized by reduced *Faecalibacterium prausnitzii* and *Lachnospiraceae* family and an increase in *Bifidobacterium*, have been recently demonstrated in patients with enthesitis-related arthritis.^[9]^ All published microbiome studies in AS are summarized in Table 4.

### Limitation of microbiome association studies

Although microbiome profiling studies have highlighted an important role of the microbiome in the pathogenesis of autoimmune diseases, it is important to highlight that there are some limitations to these studies. First, majority of microbiome studies have profiled microbiota at single time point and lack functional studies to determine the mechanism through which gut microbiota might predispose/worsen or protect from disease. It is also not clear whether microbiome is the cause of the disease or effect of the disease. Besides, there are technical challenges as there are no standardized methods for performing microbiome studies specifically in regard to sample collections, storage, DNA extraction, and library preparation, including selection of 16s primers.^[38,39]^ These technical limitations combined with the fact that as little as 10% of taxa might be shared across a given population make it difficult to perform intra- and inter-studies comparison. In addition, the bioinformatics pipelines used for microbiome analysis and statistical test(s) being used also vary among studies. Recently, it has been shown that classifying bacteria into Operational Taxanomic Unit (OUT) using 97% homology is not accurate and is slowly being replaced with amplicon sequence variant-based taxonomic classification.^[40,41]^ In addition, bacterial abundances need to be analyzed using optimal and appropriate statistical tests similar to gene expression analysis by accounting for multiple comparisons. Despite these challenges, microbiome studies clearly suggest an important role of the microbiome in health and disease and studies are underway to determine the potential mechanism through which gut microbiota can influence immune disorders.

## Outside in or Inside out: Mechanisms through Which Gut Dysbiosis Influences Autoimmune Disorders

Microbiome studies across multiple autoimmune disorders strongly suggest a role of gut microbiome in predisposition and/or propagation of disease. However, the precise mechanisms through which gut microbiota modulate host immune system to influence disease outcomes are still being investigated. The major question in the field is: if there exists direct or indirect evidence of causality between oral and gut microbiome and disease phenotypes. This has enabled us to design “Inside out versus Outside in” hypothesis. Specifically, whether oral and/or gut microbial dysbiosis is responsible for disease initiation (inside out) or inflammation during autoimmune disorders leads to gut dysbiosis (outside in). As autoimmune processes are initiated well in advance, even before the first manifestation of clinical disease, it would be prudent to refrain from assigning causality until longitudinal studies are performed. However, indirect evidence (s) from clinical studies and animal models of autoimmune disorder suggests a potential causative role of gut microbiome. We discuss the potential mechanisms through which gut bacteria and/or their metabolites can influence the autoimmune disorders.

The adult human gut is colonized by a large number of microorganisms (^~^10^13^ bacteria). The majority of which (^~^90%) belong to the *Firmicutes* and *Bacteroidetes* phyla.^[12]^ The remainder belongs to *Actinobacteria*, *Proteobacteria*, and few other phyla present at very low abundance. The presence of only few selected bacterial phyla in the human gut points toward an active selection of bacterial community during human evolution.

In addition, these selected gut and oral bacteria help the host with various physiologic functions, including the development and regulation of immune system. Bacteria can influence host immune system either directly or indirectly through the production of metabolites. Gut bacteria such as segmental filamentous bacteria (SFB) can directly adhere to intestinal epithelial cells (IECs) in the ileum and promote generation of T-helper (Th) 17 cells.^[42]^ During normal homeostasis, Th17 cells are required for clearing extracellular infections; however, the same Th17 cells have been linked with majority of inflammatory diseases including RA, MS, AS, and lupus.^[43–47]^ In contrast, due to thick mucus layer in the colon, bacteria generally influence immune system indirectly through the production of metabolites including generation of FoxP3+CD4+regulatory T (Treg) cells.^[48–51]^ A number of these metabolites such as short chain fatty acids (SCFAs), equol, serotonin, kynurenine, indole, and retinoic acids are produced by action of microbial community.^[14,21,52,53]^ Bacteria are separated from the host by single cell epithelial cell barrier which allows selective passage of metabolites from gut lumen into systemic circulation. The tight junctions between intestinal/oral epithelial cells play an important role in preventing bacteria from translocating into the extraluminal space of the gut.^[54]^ During normal homeostasis, the diverse bacterial repertoire (symbionts) enables and assists in optimal nutrient absorption, training/shaping of immune system, and preventing (physical) colonization by pathogenic bacteria (pathobionts). Thus, any perturbations in healthy bacterial (symbionts) composition can lead to colonization/expansion of pathobionts, which is defined as gut dysbiosis. This can lead to a cascade of events leading to establishment of an inflammatory environment, which can promote autoimmune disorders. First, gut dysbiosis can lead to the disruption of tight junctions between IECs, leading to increased gut permeability and translocation of bacterial products into systemic circulation.^[55,56]^ This can induce/activate innate immune cells which consequently will activate and propagate autoreactive T and B cells responses. Activation of autoreactive immune (T/B) lymphocytes can occur either directly through molecular mimicry (bacterial antigens cross-reacting with autoantigens) or through the influence of metabolites that are produced because of gut dysbiosis. However, certain microbial flora can be protective too as gut bacteria help in producing beneficial metabolites such as SCFA and equol by digesting dietary compound.^[14,21,53]^ As patients with autoimmune disorders show depletion of certain bacteria, gut/oral bacteria, and/or their metabolites, we believe that replenishment of symbionts can be used as therapeutic tool in altering disease outcomes and treating autoimmune disorders.

### Eubiosis, dysbiosis, and energy homeostasis

Having higher cells and genes than the host, gut microbiomes specifically bacteria play an important role in host physiology including efficiently energy extraction. A diverse and balanced bacterial community during the healthy state of the host is called eubiosis. A significant change in the microbiome community structure based on the diet strengthens the idea that gut microbiota can have strong effect on host energy homeostasis. Just after birth when infants are on milk-rich diet, they mostly harbor *Proteobacteria* such as *Bifidobacterium* and *Firmicutes* such as *Lactobacillus*. A change to solid diet results in gradual loss of *Proteobacteria*, expansion of *Bacteroidetes* and *Firmicutes* phyla, plus proliferation of IECs. During adulthood, *Bacteroidetes* and *Firmicutes* constitute majority of gut microbial community and accompanied with lengthening of the intestine which helps in efficient absorption of digested food. Thus, gut microbial community plays a crucial role on maintaining homeostasis at mucosal surfaces. Any perturbation of this environment by factors such as dietary changes, stress, chemicals, and antibiotics can alter this well-balanced microbial community, leading to dysbiosis. Dysbiosis can be defined as expansion of pathobionts, and depletion of symbionts is linked to the number of pathological conditions. Thus, dysbiosis is an important investigation in microbiome research in an attempt to understand the mechanism through which altered bacterial community might be involved in the pathogenesis of autoimmune diseases. Importance of microbiome in the energy homeostasis is best highlighted by the study showing that gut microbiome from obese individuals has increased capacity to harvest energy as fecal microbiota transplant from obese but not lean twins leads to weight gain in germ-free (GF) mice.^[57]^ Transplantation of gut microbiota from obese mice (high-fat diet fed) to lean GF mice resulted in higher fat deposition than a microbiota transplant from lean mice.^[58]^ The ability of the microbiome from diet-induced obese mice to cause obesity in a lean GF mouse was hypothesized to be due to the increased energy-harvesting capacity of bacteria linked with obese microbiota.^[59]^ Association of obesity as a risk factor for multiple inflammatory diseases together with the ability of microbiome to influence obesity^[60]^ emphasizes the importance of gut microbiome in the regulation of energy homeostasis.^[61]^ Thus, dysbiosis can disturb the host energy homeostasis and resulting imbalance can promote proinflammatory environment, leading to predisposition/progression of autoimmune diseases. However, the field is still evolving on the precise role of gut microbiome in energy homeostasis.

#### Dietary metabolites influencing anatomy and physiology of the gut

During homeostasis, symbionts (beneficial bacteria) maintain balance between pro- and anti-inflammatory immune response toward anti-inflammatory through conversion of ingested food (fiber, polyunsaturated fatty acid containing food, tryptophan, and phytoestrogens) into metabolites such as SCFA, Specialized proresolving lipid mediator (SPM) precursors (ω 3 fatty acids), indoles, and equol.^[14,21,52,53,62]^ These beneficial metabolites help in maintaining an anti-inflammatory milieu at the mucosal surfaces through multiple mechanisms, including the induction of immunoregulatory cells.^[14]^ Diet has the most significant influence on shaping gut microbial community.^[63]^ Thus, gut microbiota can play an important role in maintaining intactness of the epithelial barrier through the production of beneficial metabolites, and on the flip side, depletion of these metabolites in patients with active autoimmune disorders strengthens the role of gut dysbiosis in autoimmune disorders.^[21,53,64]^

#### Leaky gut syndrome

**Figure 1:**
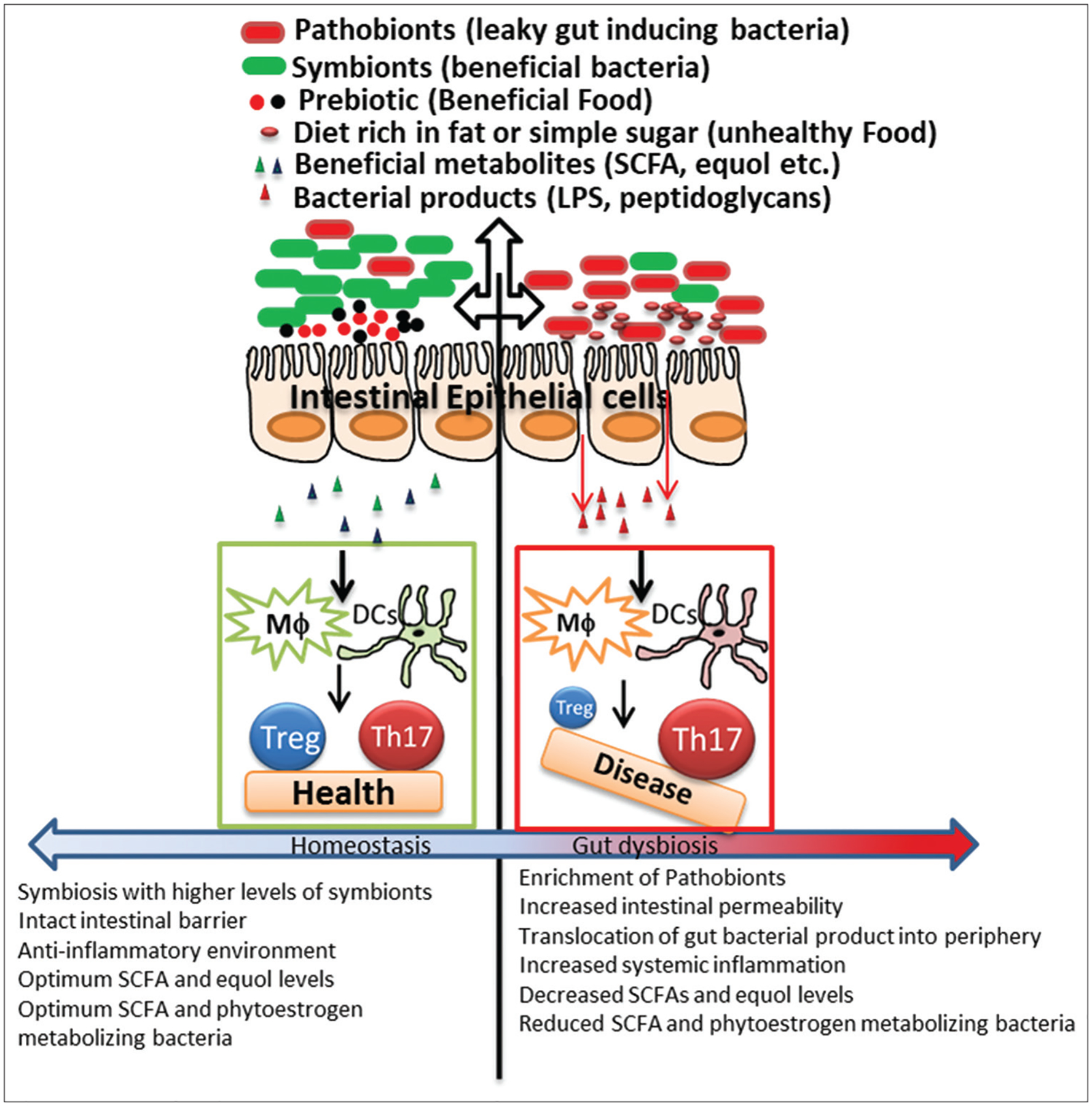
Leaky gut syndrome. During a healthy state, there is a diverse gut microbial community (microbiome) which helps in maintaining the homeostasis at mucosal surfaces. Beneficial bacteria metabolize dietary components such as diet-containing fiber and/or phytoestrogens into health-promoting metabolites such as short chain fatty acids and equol. These beneficial metabolites help in creating tolerogenic immune environment by maintaining a balance between anti-inflammatory regulatory T cells and proinflammatory T-helper 17 cells

Alterations in the composition of gut microbiota with enrichment of pathobionts and depletion of beneficial symbionts are linked with gut barrier dysfunction. Increased intestinal permeability (leaky gut) will also enable translocation of bacterial endotoxins such as lipopolysaccharide (LPS) into systemic circulation, and LPS is a potent inducer of proinflammatory mediators.^[65]^ The processes of leaky gut and systemic inflammation, induced by increased levels of leaky gut inflammatory mediators, are termed leaky gut syndrome [Figure 1], which had been linked with multiple inflammatory diseases, including MS.^[66]^ It is also believed that gut microbiota induce the activation of zonulin pathway, which is integral in the maintenance of epithelial tight junctions. Enhanced tissue levels of zonulin were demonstrated in AS ileal samples and accompanied by a profound reduced expression of tight junction proteins by the epithelial cells.^[66]^ It is not clear if tight junction alterations are a consequence of gut dysbiosis. Oral antibiotic treatment in HLA-B27 transgenic rats alters gut microbial flora and also reduces epithelium adherence of bacteria, suggesting that intestinal dysbiosis might be confounding integrity of the epithelial barrier.^[67]^ The gut vascular barrier has also gained prominence in recent times.^[68]^ The presence of a “leaky endothelium” was observed in patients with AS where disorganized staining was observed for claudin, occludin, and zonulin and was accompanied by increased serum levels of zonulin and bacterial products such as LPS, LPS-binding protein (BP), and intestinal fatty acid-BP.^[69]^ The presence of increased circulating levels of bacterial products in AS is accompanied by the downregulation of CD14 on the surface of monocytes together with the reduced expression of HLA-DR proteins.^[70]^ Despite the evidence suggesting presence of dysbiosis and functional relevance of dysbiosis in patients with AS, the use of probiotics had not demonstrated to significantly modulate the disease activity in AS patients. These observations suggest that once the inflammatory process has been established, it proceeds independently of the bacterial stimulus.^[71]^ Altogether, these studies support the occurrence of dysbiosis in patients with SpA, thus highlighting the role of HLA-B27 in shaping intestinal microbiome.

#### Molecular mimicry

One of the mechanisms through which bacteria can influence inflammation is through molecular mimicry where bacterial antigens have similarity to disease-specific autoantigens. Autoreactive T and/or B cell activation can occur in a bystander manner during a nonspecific inflammatory response, resulting in organ-specific inflammation. This inflammatory cascade could further perpetuate and lead to release of autoantigens secondary to organ-specific inflammation and add fuel to the fire.

Autoantibodies against the Ro60 antigen are considered pathognomonic and known to occur in susceptible individuals before the development of SLE clinical syndrome.^[72]^ It is very well accepted that environmental triggers such as sunlight lead to exacerbation of disease in SLE-susceptible individuals. It has been hypothesized that susceptible individuals with a specific HLA-haplotype cross-react with microbial antigens that are similar in sequence or structure to the Ro60 autoantigen. Peptide cross-reactivity with Ro60 epitopes targeted in SLE patients was shown with a murine T cell hybridoma.^[73]^ Greiling TM *et al*. have identified cross-reactive responses between human gut, oral, and skin microbiota from SLE patients and human Ro60 autoantigen.^[72]^ Since Ro60 ortholog-expressing bacteria chronically colonize skin and mucosal sites of genetically predisposed hosts, cross-reactive responses are likely to initiate and propagate autoimmunity *in vivo*. Interestingly, *Propionibacterium propionicum*, which was detected in the skin lesions of patients with subacute cutaneous lupus erythematosus, also has a Ro60 ortholog. Furthermore, *Corynebacterium amycolatum* has been shown to colonize the lacrimal duct and could therefore be involved in the pathogenesis of dry eye symptoms in anti-Ro60–positive antibodies in Sjögren’s syndrome patients.^[74]^ These studies in humans are intriguing and highlight the role of molecular mimicry in inducing immune responses which are associated with SLE and Sjögren’s syndrome.

Similarly, in MS, the number of microbial pathogens has been proposed to have proteins which have similarity to myelin antigens. Numerous studies have shown that Epstein–Barr virus antigens are cross-reactive with myelin-specific CD4 T, CD8 T, and B cells.^[75–79]^ However, disease-initiating antigens might not be myelin specific as shown by the recent study by Planas R *et al*.^[80]^ They used brain-infiltrating CD4 T cell clones from MS patients to identify guanosine diphosphate-L-fucose synthase as a potential autoantigen which was cross-reactive with myelin antigen. Most importantly, these autoreactive CD4 T cells can also be activated by homologous peptide from gut microbiota, especially from *Akkermansia*. This study suggests that gut microbiota-derived protein can also directly stimulate autoreactive T cell repertoire.

Role of gut bacterial-derived antigen as a potential mimic for inducing RA is understudied. Few studies have predicted that gut bacteria such as *Clostridium, Eggerthella, Bacteroides* and *Citrobacter atopobium, Oribacterium, Actinomyces,* and *Cryptobacterium* mimicked motifs of collagen XI and HLA-DRB1*0401.^[24,81]^ It is well known that oral cavity and dental caries are caused by *Porphyromonas gingivalis*. At the periodontal level, *P. gingivalis* has peptidylarginine deiminase (PAD) enzymes and these enzymes alter self-proteins through posttranslational modifications. One such posttranslational modification is citrullination, the conversion of arginine residues to citrulline by PAD enzymes. Moreover, through gingipains, *P. gingivalis* increases the polarization of T cells toward Th17. Novel citrullinated peptides are preferentially bound by HLA-DR-B1 shared epitope alleles, such as HLA-DR*0401 (HLA-DR4).^[82]^ This leads to the activation of antigen-presenting cells and induction of anti-citrullinated protein immune responses. Detection of ACPAs is a hallmark and is a diagnostic criterion for RA. ACPA targets include filaggrin, collagen, α-enolase, fibrinogen, and vimentin and are used as specific markers to diagnose RA. It is very well documented that predisposed individuals have the presence of ACPA antibodies few years before the development of RA disease phenotype, and there is a progressive increment in the titers of ACPA antibodies in the serum of these individuals and higher ACPA titers are associated with severe RA, thus having a diagnostic and phenotypic influence. These posttranslational modifications are initiated by PAD enzymes from *P. gingivalis* a pathobiont of oral cavity in RA patients with active disease.

#### Induction of proinflammatory T-helper 17 response by gut bacteria

In the last two decades, Th17 CD4^+^ T cells have emerged as a major pathogenic cells in autoimmune diseases.^[48–51]^ As discussed above, microbiota play a critical role in the generation of these Th17 cells.^[42,83,84]^ Importance of gut microbiota in the generation of Th17 cells can be highlighted by the fact that there is absence or severely reduced levels of IL-17 in GF mice.^[42]^ Animal models of human mono/poly-genic autoimmune disorders are influenced by microbiota. Murine models of T1D, i.e., nonobese diabetic mice and BB rats, develop spontaneous autoimmunity when harbored in controlled (sterile) environments.^[85]^ On the other hand, murine models of RA are known to have exacerbations of clinical disease when raised in (nonsterile) environments. These results highlight the ability of microbiota to induce disease states. Mono-colonization with SFB augmented RA in a mouse model^[86]^ however had no effect in the context of T1D.^[87]^ These studies suggest that different microbes can be protective, neutral, or provocative for the development of autoimmunity.

SFB has been also shown to play a role in the pathogenesis of experimental autoimmune encephalomyelitis (EAE), animal model of MS, and its ability to induce proinflammatory response, i.e., specially Th17 cells are required for the development of disease in animal model of MS.^[88]^ Importance of gut microbiota in the development of disease in animal model of MS is well documented. Ochoa-Repáraz *et al*. were the first to show the importance of gut microbiota in animal model of MS as the removal of gut microbiota by the treatment with broad-spectrum antibiotic led to the impairment of disease development.^[89]^ Similarly, Lee *et al*. showed that GF mice develop attenuated EAE.^[88]^ However, colonization of GF mice with SFB led to the development of severe EAE, and the ability of SFB to induce IL-17A–producing CD4 T cells played a critical role in the development. Presence of SFB also leads to proinflammatory dendritic cells (DCs) with better antigen presentation capacity. Importance of gut microbiota in the development of EAE was further shown by Berer *et al*. utilizing GF Swiss Jim Lambert (SJL) mice expressing Myelin oligodendrocyte glycoprotein (MOG)-specific CD4 T-cell receptor (TCR).^[90]^ While these TCR^tg^ GF mice were protected from EAE, colonization with gut microbiota from conventional mice led to the development of severe EAE as in conventional colonies. The reduced disease was due to impaired T and B cell activation, especially production of IL-17 and MOG-specific antibody, respectively. Interestingly, in this study, SFB alone failed to activate Th17 for full-blown disease. Thus, both Lee *et al*.^[88]^ and Berer *et al*.^[90]^ have suggested an important role of gut microbiota in disease development; however, the discrepancies between two studies indicate that it is not a specific bacterium, but any gut bacteria with the ability to stimulate Th17 cells can trigger autoimmune disease.

### Microbial modulation as therapy to treat autoimmune diseases

Autoimmune diseases are only observed in a very small fraction of any given population. Taking into consideration the existing data on microbiome signatures in healthy individuals, it is fair to postulate that microbiota predominantly have a protective role, thus enabling us to harness the power of microbiome and utilize them as potential therapeutic agents to treat various autoimmune diseases. Number of groups including ours is working on this front. The gut commensal-based therapies can be divided into two groups; first, use of probiotics and second, bacteria as drugs (BRUGs). We have coined the term BRUG to differentiate it from probiotics as the latter are harmless bacteria which may or may not be reduced during gut dysbiosis. Besides probiotics and BRUG, gut microbial modulation can be achieved through prebiotics (diet) or fecal transplantation.

#### Probiotics-based therapy to treat autoimmune diseases

According to the National Center for Complimentary and Integrative Health, USA, probiotics are defined as live organisms which can provide health benefits when consumed or applied to the body. A number of probiotic mixtures are currently being tried as a therapy for various autoimmune diseases; however, at present, there is no consensus on the efficacy of probiotics in MS, RA, T1D, lupus, or AS. Hatakka *et al*. studied the effects of probiotics *Lactobacillus rhamnosus* GG (LGG) on RA patients from Finland in a double-bind fashion for 12 months.^[91]^ Although there was no significant difference in overall clinical parameters, inflammatory mediators, and health assessment questionnaire index,^[91]^ the group receiving LGG reported feeling better. In an Iranian study, Zamani *et al*. have analyzed the effect of probiotic mix containing mixture of *Lactobacillus acidophilus*, *Lactobacillus casei*, and *B. bifidum* for 8 weeks in RA patients in a randomized, double-blind, placebo-controlled trail.^[92]^ RA patients receiving probiotic supplementation showed improved disease activity score (DAS)-28 and high-sensitivity C-reactive protein (hs-CRP) levels. Another study from Iran also showed that supplementation with *L. casei 01* alone resulted in improved DAS-28 and reduced levels of hs-CRP.^[93]^ However, a recent meta-analysis of nine randomized control trials on the therapeutic effect of probiotics in RA reported that probiotics supplementation did not cause any significant difference in DAS compared to placebo group.^[94]^ In addition, Liu *et al*. showed that RA patients have higher abundance of *Lactobacillus* genera compared to HC, suggesting that either *Lactobacillus* is pathogenic in RA or even high levels of *Lactobacillus* fail to suppress RA. Several studies have tested ability of probiotics (strains of *Bifidobacterium, Lactobacillus, Clostridia, Ruminococcus, and Synergistetes*) for immune modulation in SLE which has been summarized in a review by Esmaeili *et al*.^[95]^ Briefly, probiotic supplementation in SLE patients was able to induce expansion of Treg cells, reduce levels of proinflammatory cytokines, especially IL-6 and Th17 cytokines, and suppress *ex vivo* CD4 T cell proliferation. Although the role of gut microbiota in the pathogenesis of MS is increasingly appreciated, few studies on testing therapeutic effects of probiotics in MS patients have been inclusive. In a randomized double-blind placebo-controlled clinical trial of Iranian MS patients, Kouchaki *et al*. showed that MS patients taking probiotic mix containing mixture *of L. acidophilus, L. casei,* and *B. bifidum* showed an improvement in Expanded Disability Status Scale (EDSS) score and inflammatory markers.^[96]^ A recent study from Weiner *et al.* showed that 8-week treatment with VSL3 containing four strains of *Lactobacillus*, three strains of *Bifidobacterium*, and one strain of *Streptococcus* modulated gut microbiota^[97]^ and induced an anti-inflammatory immune response. However, this study did not report any effect of probiotics on improvement of MS disease activity itself. There is only one randomized controlled trial on the use of probiotic therapy for the treatment of AS. Sixty-three AS patients were randomized to receive either probiotic mix containing *Streptococcus salivarius*, *Bifidobacterium lactis* LAFTI B94, and *L. acidophilus* or placebo.^[98]^ However, probiotic therapy showed no benefits over placebo. In summary, the effect of probiotic therapy in autoimmune diseases is still inconclusive, and future studies with different probiotic combination and/or longer duration might provide an answer on effectiveness of probiotics for the treatment of autoimmune diseases.

#### Bacteria as drugs

As probiotics-based therapy has been inconclusive in the treatment of autoimmune diseases, a number of groups including ours have focused on the utilization of mono-colonization with single bacterium, specifically those lacking in the patients. Majority of probiotics used in clinical trials contain a mixture of *Bifidobacterium*, *Lactobacillus*, and *Streptococcus*; however, microbiome studies comparing patients with RA, MS, AS, and lupus have failed to report lack of these bacteria in patients compared to HCs. Thus, a better approach will be identifying the bacterium lacking specific autoimmune diseases and used them as a therapy. Our argument is that providing a specific bacterium lacking in a patient will be better at treating the disease than a nonspecific commensal. We have coined the term BRUG to differentiate it from probiotics as the later are harmless bacteria which may or may not be reduced during gut dysbiosis. We plan to utilize a specific BRUG, designed from analyzing microbial signatures, in the therapeutics of a particular autoimmune disorder. Experimental biologists and clinicians are gradually learning that one size does not fit all and therapeutic philosophy is progressing toward precision and personalized medicine. Thus, we argue that BRUG might have better therapeutic potential than one size fit all probiotics.

Multiple studies in MS have validated the importance of *Prevotella* genus as this bacterium displays reduced abundance in MS patients and increased abundance after treatment with Disease Modifying Treatmants (DMTs) [Table 1]. Based on these, we were successful in isolating a strain of *Prevotella* and *Prevotella histicola* from human subjects which suppressed disease in animal model of MS.^[6,99,100]^ We also reported that *P. histicola* was efficient in suppressing disease in EAE as currently used first-line MS therapies copaxone^[100]^ and betaseron (manuscript under review). Interestingly, *P histicola* was also able to suppress disease in collagen-induced arthritis model in HLA-DR-4 transgenic mice^[101]^ and T1D mouse model (manuscript under review).

There are other microbiota candidates such as *Bacteroides fragilis* which have been shown to suppress disease in animal models of multiple diseases including MS,^[102]^ colitis,^[103]^ and autism spectrum disorders.^[104]^ This was the first gut commensal which has been proposed by Mazmanian *et al*. as potential therapeutics. Studies from Mazmanian *et al*. as well as others have published extensively on therapeutic ability and potential mechanism of *B. fragilis* in modulating immune response,^[105]^ preventing colonization of pathobionts, and suppressing inflammatory and neurological diseases including MS, IBD, and autism spectrum disorders. Mazmanian *et al*. showed that *B. fragilis* can help in the maturation of host immune system and immunomodulatory ability of *B. fragilis* was dependent on bacterial polysaccharide (PSA).^[105]^ The mutant *B. fragilis* lacking PSA failed to direct maturation of host immune response. This group next showed that PSA-expressing *B. fragilis* but not the mutant lacking PSA can prevent the development of colitis^[103]^ and EAE.^[102]^ Ability of PSA to suppress disease was due to its ability to induce FoxP3^+^ CD^+^ regulatory T cells,^[106]^ as well as IL-10–producing B and T cells.^[103,107]^ Thus, all these studies highlight the ability of *B. fragilis* to perform immunomodulatory function and its potential as a therapeutic agent to treat inflammatory autoimmune diseases.

#### Prebiotics/diet-based therapy

Prebiotics are foods that are degraded by gut bacteria into metabolites which are beneficial to human health.^[108]^ Since diet has the strongest impact on gut microbiome even more than genetic effect,^[109]^ it is not surprising that scientific community is slowly becoming more receptive to diet as a potential therapeutic agent. Importance of diet in human diseases can be highlighted by multiple clinical trials undergoing based on diets such as fasting-mimicking diet, Mediterranean diet, Keto diet, Paleo diet, and Wahls diet.^[110–117]^ As this is an emerging field and multiple clinical trials are underway, we need to wait for few years before discussing the effectiveness of diet-based therapies. However, interested readers are encouraged to read excellent reviews mentioned above.

### The influence of HLA-type on microbiome in rheumatic diseases

Among all the genetic factors linked with autoimmune diseases, *HLA* genes on chromosome 6 show the strongest association with susceptibility versus resistance to autoimmune diseases.^[118]^ HLA molecules help in shaping the diverse and strong adaptive immune response required for tackling a variety of pathogens we face every day. Thus, in majority of the population, HLA molecule-induced immune responses help us survive attack by pathogens; however, in certain individuals, disease-susceptible HLA molecules can predispose to the development of autoimmune diseases. Autoimmunity is believed to be unwanted and adverse effect of having a robust immunity. As microbiome(s) also regulate host immune responses, it is not surprising to hypothesize that HLA can influence the host microbiome. Although HLA and microbiome have been individually linked with majority of autoimmune diseases, the importance of HLA in influencing microbiome in the pathobiology of autoimmune disease is best highlighted by studies performed in SpA microbiome.^[4,119,120]^ HLA-B27 had been established as an important risk factor for the development of SpA, and the number of studies in animal models had proved the same. Requirement of both HLA and gut bacteria in the development of SpA is highlighted by studies utilizing *HLA-B27* transgenic rat.^[121–123]^ Specifically, transgenic rats expressing human HLA-B27 molecules develop SpA-like disease only when exposed to specific pathogen-free enteric bacteria but not in the GF facility.^[123]^ In human studies, colonic microbiome of HLA-B27–positive SpA patients was different from B27-negative SpA patients.^[4]^ Association between HLA-allele and microbiome has been also shown in RA, Celiac sprue, and Crohn’s disease.^[124–127]^ Presentation of bacterial antigen by disease-susceptible HLA molecules is one of the possible mechanisms through which HLA can collude with gut bacteria to cause disease (discussed in detail under molecular mimicry section). Utilizing transgenic mice expressing either RA-susceptible HLA_ DRB1*0401 or RA-resistant DRB1*0402, Gomez *et al*. showed that these two alleles of HLA-DR-4 selected different microbiome.^[128]^ Thus, above studies highlight that modulation of gut microbiota by host HLA molecules can predispose to or protect from autoimmune disease. However, more detailed studies are required to understand the detailed mechanism (s) through which HLA molecules can affect gut microbiota as well as whether gut bacteria or bacterial products can modulate expression of HLA molecules.

### Conundrums

The world of “Microbiome Biology” has exploded in the last decade and has gained significant attention from clinicians and experimental biologists. It is evident from the data that pathobionts are associated with disease states and symbionts with health. Precise mechanisms that enable pathobionts to influence disease outcomes are being studied by various groups including ours. A given microbiome signature is unique to a given individual; however, there are similarities in the microbiome signatures within a given community. Microbiome signatures display a spectrum of core members during steady states. However, during disease state, there are fringe members (pathobionts) that invade the microbial community, disrupt the organization, and encroach and take over a significant space (quantitative abundance), which results in gut dysbiosis. Corollaries can be drawn in social structures of communities when fringe members (vagabonds) invade certain communities and disrupt social harmony that exists within that community. To revert back to a healthy state in their respective communities, these pathobionts (vagabonds) need to be replaced by symbionts (wise people) for that individual and community to thrive and prosper. Complexities of these interactions are displayed in another piece of evidence that symbionts can become pathobionts in given individuals. It is well known that the depletion of *Akkermansia muciniphila* has been noted in individuals with type-2 diabetes mellitus (DM) and obesity.^[129–131]^ Furthermore, replenishment of *A. muciniphila* in mice with type-2 DM ameliorated disease development. This notwithstanding, *A. muciniphila* is considered a pathobiont in MS as MS patients have higher abundance compared to HCs.^[8,17]^ These data collectively intrigue us to postulate that there is a plethora of factors such as tissue tropism, influence of community members, and qualitative changes within microbes (symbionts and pathobionts) that contribute to gut dysbiosis. The later has been dissected out by elegant studies which highlight the importance of acyl chains on the LPS molecule of Gram-negative bacteria.^[132]^ LPS engages conserved receptors (Toll-like) that lead to immune activation to clear infection and maintain homeostasis. However, in certain clinical situations, the same LPS can lead to hyper-immune activation leading to multiorgan dysfunction. First-degree relatives of SLE patients have a higher level of circulating and soluble LPS in the serum which also corresponds to the autoantibody levels of anti-dsDNA.^[133]^

It is evident from various studies that specific microbes are enriched during certain pathological states. It is also well known that many autoimmune disorders undergo exacerbations and reemissions with seasonal changes. Although these anecdotal beliefs held in the field of autoimmunity, there is a paucity of studies that provide conclusive evidence, highlighting the role of seasons (winter/summer/fall) on autoimmune disease progression. This begs us to ask the question, “If there exists a link between seasonal variations and microbiome signatures, and furthermore, if there is a correlation with autoimmune disease exacerbation/remission.” This would give credence to the “outside in” hypothesis where the environment would influence the gut microbial repertoire present inside the luminal surfaces. To dissect this hypothesis, longitudinal analysis of microbial signatures in vulnerable cohorts will be warranted and needs to be undertaken.

Most autoimmune disorders are noted to have varying disease penetrance and hence have a spectrum of clinical manifestations within the broad disease-specific diagnosis. For example, some of the patients with SLE can have skin lupus while others can have lupus nephritis. Varying microbial signatures are known to be associated with skin lupus versus lupus nephritis,^[134]^ thus highlighting the importance of variability of microbial signature within a given autoimmune disorders. This intrigues us to address the question if microbial signatures govern clinical outcomes or/if clinical syndromes influence microbial signatures. The variability of microbial signatures noted within a clinical syndrome highlights the importance of developing management strategies to personalize therapeutic options. We have coined the term BRUGs which will be utilized in the current therapeutic modalities as synergistic molecules to treat autoimmune disorders. This also highlights the importance of clinicians, basic scientists, and nutritionists working as a team, to better define the role of microbiome in health and disease. This will also allow to harness the BRUG as the potential therapeutic treatment option.

## Conclusions

As our understanding of microbial variation and corresponding genetic parameters continues to grow, remodeling of the human gut microbiota and their associated functions has attained an intriguing milestone in the development of therapeutic options for various autoimmune disorders. Data from prospective, randomized, multicenter, longitudinal studies are still awaited to enables us to prove the success of microbiota-targeted approaches to treat various autoimmune disorders. In an ideal setting, personalized strategy can be designed which will enable the team consisting of clinician, basic scientist, and nutritionist to develop dietary intervention strategies, which will favor the increase of specific microbial populations that, perhaps, could have an influence on autoimmune disorders. These may be in the form of custom diets and/or probiotics or targeted antibiotics. The utilization of such treatments, in individual or combined regimens, may manipulate the gut microbiota in a way that will prove to be a “game-changer” in modern medicine. Although this sounds simple, there is still a need to carefully investigate the complex interactions in the context of host genetic backgrounds to identify the optimal treatment strategies. However, the complexity of interactions between the host and the gut microbiota still warrants the question of whether therapeutic interventions will be successful for a majority of the individuals suffering from this complex disease of immune dysregulation and this question has yet to be answered.

## Figures and Tables

**Table 1:** Comparison of adult multiple sclerosis microbiome studies

MS microbiome study # samples Tissue (Country)	Lower abundance in MS patients versus HC	Increased abundance in MS patients after treatment
RRMS (*n*=31) HC (*n*=36) Fecal (USA)^[6]^	*Prevotella, Parabacteroides, Adlercreutzia, Collinsella, Lactobacillus*	
RRMS (*n*=60) HC (*n*=43) Fecal (USA)^[8]^	*Butyricimonas, Prevotella, Parabacteroides*	*Prevotella* *Sutterella*
RRMS (*n*=20) HC (*n*=40) Fecal (Japan)^[18]^	*Bacteroides, Faecalibacterium, Prevotella, Anaerostipes, clostridium, Sutterella*	
RRMS (*n*=30) HC (*n*=14) Fecal (UK)^[19]^		*Prevotella*
RRMS (*n*=71) Fecal (USA)^[17]^	*Parabacteroides distasonis*	
RRMS (*n*=19) HC (*n*=17) Mucosa (Italy)^[20]^	*Prevotella*	

MS: Multiple sclerosis, HC: Healthy control, RRMS: Relapsing and remitting multiple sclerosis

**Table 2:** Comparison of adult rheumatoid arthritis microbiome studies

RA microbiome Study # samples Tissue (country)	Lower abundance in RA patients versus HC	Increased abundance in RA patients versus HC
RA (*n*=42) HC (*n*=10) Fecal (Italy)^[23]^	*Faecalibacterium, Flavobacterium, Blautia*	*Lactobacillus*
RA (*n*=40) HC (*n*=32) Fecal (USA)^[7]^	*Faecalibacterium*	*Actinobacteria, Collinsella, Eggerthella, Actinomyces*
NORA (*n*=44) HC (*n*=28) (USA)^[25]^	*Bacteroides*	*Prevotella copri*
RA (*n*=77) HC (*n*=80) Fecal (China)^[24]^	*Haemophilus spp., Veillonella, Klebsiella pneumoniae, Bifidobacterium bifidum, Sutterella wadsworthensis, Megamonas hypermegale*	*Lactobacillus, Eggerthella, Clostridium asparagiforme, Gordonibacter, Pamelaeae, Lachnospiraceae bacterium*
RA (*n*=21) HC (*n*=23) Fecal (Canada)^[22]^	*Roseburia, Gemmiger, Lachnospira, Sporobacter*	*Actinomyces, Eggerthella Clostridium III, Faecalis coccus Streptococcus*
RA (*n*=110) HC (*n*=155) Oral (China)^[5]^		*Neisseria subflava, Haemophilus parainfluenzae, Veillonella dispar, Prevotella tannerae, Actinobacillus parahaemolyticus, Neisseria, Haemophilus, Prevotella, Veillonella, Fusobacterium, Aggregatibacter, Actinobacillus*

RA: Rheumatoid Arthritis, HC: Healthy control

**Table 3:** Comparison of adult Systemic lupus erythematosus microbiome studies

SLE microbiome study # samples Tissue (country)	Lower abundance in SLE patients versus HC	Increased abundance in SLE patients versus HC
SLE (*n*=20) HC (*n*=20) Fecal (Spain)^[26]^	*Firmicutes*	*Bacteroidetes*
SLE (*n*=45) Fecal (China)^[27]^	*Dialister, Pseudobutyrivibrio*	*Rhodococcus, Eggerthella, Klebsiella, Prevotella, Eubacterium, Flavonifractor*
SLE (*n*=14) Non-SLE control (*n*=17) Fecal (USA)^[28]^	*Odoribacter*	*Proteobacteria, Blautia*
SLE (*n*=61) Fecal (USA)^[29]^		*Ruminococcus gnavus*
SLE (*n*=52) HC (*n*=52) Oral (Brazil)^[30]^		*Fretibacterium, Prevotella nigrescens, Selenomonas*
SLE (*n*=20) HC (*n*=19) Oral (China)^[31]^	*Sphingomonadaceae, Halomonadaceae, Xanthomonadaceae*	*Lactobacillaceae, Veillonellaceae, Moraxellaceae*

SLE: Systemic lupus erythematosus, HC: Healthy control

**Table 4:** Comparison of adult ankylosing spondylitis microbiome studies

AS Microbiome Study # samples Tissue (country)	Lower abundance in AS patients	Increased abundance in AS
AS (*n*=9)	*Veillonellaceae*	*Lachnospiraceae*
HC (*n*=9)	*Prevotellaceae*	*Porphyromonadaceae*
Terminal ileum		*Ruminococcaceae*
Biopsy specimen^[33]^		*Bacteroidaceae*
		*Rikenellaceae*
SpA (*n*=87)	*Prevotellaceae*	*Ruminococcus gnavus*
RA (*n*=28)		
HC (*n*=69)		
Fecal (France)^[4]^		
AS (*n*=73) HC (*n*=83) Fecal (China)^[34]^	*Bacteroides* spp.	*Prevotella melaninogenica, Prevotella copri, Prevotella* sp.
AS (*n*=41) HC (*n*=21) Fecal (China)^[35]^	*Eubacterium ruminantium, Ruminococcus gnavus, Lachnospira, Bacteroides*	*Prevotella 9, Dialister, Comamonas, Collinsella, Streptococcus, Alloprevotella, Prevotella 2*
AS (*n*=150) HC (*n*=17) Fecal (Sweden)^[36]^	*Bacteroides, Lachnospiraceae*	*Proteobacteria, Enterobacteriaceae, Bacilli, Streptococcus species, Actinobacteria*
AS (*n*=85) HC (*n*=62) Fecal (China)^[37]^		*Bacteroides coprophilus, Parabacteroides distasonis, Eubacterium siraeum, Acidaminococcus fermentans, Prevotella copri*

As: Ankylosing spondylitis, HC: Healthy control, SpA: Spondyloarthropathy, RA: Rheumatoid arthritis
